# Hair cortisol-a stress marker in children and adolescents with chronic tic disorders? A large European cross-sectional study

**DOI:** 10.1007/s00787-020-01714-1

**Published:** 2021-01-18

**Authors:** Judith Buse, Josefine Rothe, Anne Uhlmann, Benjamin Bodmer, Clemens Kirschbaum, Pieter J. Hoekstra, Andrea Dietrich, Veit Roessner, EMTICS collaborative group, Alan Apter, Valentina Baglioni, Juliane Ball, Noa Benaroya-Milshtein, Benjamin Bodmer, Emese Bognar, Bianka Burger, Judith Buse, Francesco Cardona, Marta Correa Vela, Andrea Dietrich, Maria Cristina Ferro, Blanca Garcia-Delgar, Mariangela Gulisano, Annelieke Hagen, Julie Hagstrøm, Tammy J. Hedderly, Isobel Heyman, Pieter J. Hoekstra, Chaim Huyser, Marcos Madruga-Garrido, Davide Martino, Pablo Mir, Astrid Morer, Kirsten Müller-Vahl, Alexander Münchau, Peter Nagy, Valeria Neri, Thaïra J. C. Openneer, Alessandra Pellico, Kerstin J. Plessen, Cesare Porcelli, Renata Rizzo, Veit Roessner, Daphna Ruhrman, Jaana M. L. Schnell, Anette Schrag, Paola Rosaria Silvestri, Liselotte Skov, Tamar Steinberg, Friederike Tagwerker Gloor, Zsanett Tarnok, Elif Weidinger

**Affiliations:** 1 Department of Child and Adolescent Psychiatry, Faculty of Medicine, Technische Universität Dresden, Fetscherstrasse 74, 01307 Dresden, Germany; 2 Institute of Biopsychology, Department of Psychology, Technische Universität Dresden, Dresden, Germany; 3 Department of Child and Adolescent Psychiatry, University of Groningen, University Medical Center Groningen, Groningen, The Netherlands

**Keywords:** Tourette, Physiological stress marker, Psychosocial stress, Chronic tic disorders, Emotional and behavioral problems

## Abstract

**Background:**

There is clear evidence that tic disorders (TDs) are associated with psychosocial stress as well as emotional and behavioral problems. Studies have shown that individuals with TDs have higher acute physiological stress responses to external, single stressors (as reflected by saliva cortisol). The aim of the present study was to examine a physiological marker of longer-term stress (as reflected by hair cortisol concentration) in children and adolescents with TDs and unaffected siblings of individuals with TDs.

**Methods:**

Two samples of a European cohort were included in this study. In the COURSE sample, 412 children and adolescents aged 3–16 years with a chronic TD including Tourette syndrome according to DSM IV-TR criteria were included. The ONSET sample included 131 3–10 years old siblings of individuals with TDs, who themselves had no tics. Differences in hair cortisol concentration (HCC) between the two samples were examined. Within the COURSE sample, relations of HCC with tic severity and perceived psychosocial stress as well as potential effects and interaction effects of comorbid emotional and behavioral problems and psychotropic medication on HCC were investigated.

**Results:**

There were no differences in HCC between the two samples. In participants with TDs, there were no associations between HCC and tic severity or perceived psychosocial stress. No main effects of sex, psychotropic medication status and comorbid emotional and behavioral problems on HCC were found in participants with TDs.

**Conclusion:**

A link between HCC and TDs is not supported by the present results.

**Supplementary Information:**

The online version contains supplementary material available at 10.1007/s00787-020-01714-1.

## Introduction

Chronic tic disorders^1^ (TDs) are characterized by motor and/or vocal tics lasting at least 1 year with their first onset commonly occurring during childhood [1], and affecting around 0.8% of children worldwide [2, 3]. Genetic factors are important in TDs [4–6], but altered immune responses and perceived psychosocial stress may also be underlying or modifying factors [7–9].

TDs are often accompanied by a variety of psychosocial problems including social stigmatization, enhanced caregiver burden and reduced quality of life, which exposes patients with TDs to the experience of psychosocial stress [10]. Psychosocial stress manifests physiologically as an activation of the hypothalamic–pituitary–adrenal (HPA) axis. Within this multi-step biochemical pathway, several brain regions pass information about environmental stressors on to the paraventricular nucleus of the hypothalamus, which, in turn, releases the corticotropin-releasing hormone (CRH) interacting with receptors of corticotropic cells in the pituitary gland. Subsequently, adrenocorticotropic hormone (ACTH) is secreted into the blood, binds to the cortex of the adrenal glands and causes the secretion of the steroid hormone cortisol, the final key messenger in the cascade. There is some evidence from small sampled studies that patients with TDs show a stronger activation of the HPA axis compared to healthy controls, when exposed to psychosocial stress. For example, patients with TDs showed higher levels of salivary cortisol during a magnetic resonance imaging (MRI) mock scan session [11], higher levels of ACTH in the blood plasma before and after a lumbar puncture [12] and higher levels of CRH in the cerebrospinal fluid during lumbar puncture [13]. Moreover, patients with TD had marginally lower levels of salivary cortisol in the evening [11]. The authors argue that lower evening cortisol might be a result of chronic daily stress, as a lower evening cortisol profile was also found in conditions of chronic stress.

One major limitation of previous studies is that the assessed physiological stress level in patients with TDs only captured a short-term assessment of cortisol levels, i.e. changes in cortisol, ACTH and CRH levels that occur over the course of minutes to hours. However, to determine how the activation of the HPA axis may be associated with the presence of chronic tics over the course of weeks to months, it is important to assess cortisol levels reflecting those time spans. For this purpose, the measurement of cortisol concentrations in hair samples offers a promising approach [14, 15], as it provides a retrospective and longer-term assessment of cumulative cortisol levels over periods of several weeks. With this method, aberrant hair cortisol concentrations have been found in adult individuals experiencing socially or physically demanding conditions (e.g. unemployment, pregnancy) or in those with various psychiatric disorders (e.g. post-traumatic stress disorder, depression, generalized anxiety disorder), compared to unaffected controls [16–21]. Aberrant hair cortisol concentrations (HCC) were also found in children and adolescents with emotional or behavioural problems. For example, conduct problems were associated with higher HCC in middle childhood, while emotional symptoms and attention-deficit/hyperactivity disorder (ADHD) symptoms were associated with lower HCC in younger children [22, 23]. Children with TDs often have comorbid psychiatric disorders, such as ADHD, social and simple phobia, separation anxiety or oppositional defiant behavior [24]. Therefore, HCC of children with TDs may be affected by both TDs and comorbid psychiatric disorders or their interaction.

A further limitation is that reports of perceived stress and physiological measures of stress have often not been investigated concurrently [11, 12, 25]. There has been one study in children and adolescents with TDs using a physiological stress measure (saliva cortisol) and a short self-report measure of the subjective perception of psychosocial stress concurrently during situations of stress, relaxation and concentration [26]. The results demonstrated that salivary cortisol level during the stress situation was higher compared to situations of concentration. To date, a combined investigation of longer-term cortisol levels and perceived stress within patients with TDs has not yet been conducted.

The aim of our study was to compare levels of HCC as a physiological marker of longer-term stress between children and adolescents with TDs and siblings of individuals with TDs, who did not have tics. We assumed that there would be higher levels of HCC in children and adolescents with TDs compared to unaffected siblings of individuals with TDs. We investigated the relations of HCC with tic severity and perceived psychosocial stress in children with TDs and hypothesized a positive relationship. Finally, as many children and adolescents with TDs are affected by comorbid emotional and behavioral problems [24, 27] and take psychotropic medication for symptom reduction of tics [28], we were interested in their potential main and interaction effects on HCC. Based on findings for associations of saliva and hair cortisol with emotional and behavior problems [22, 29–31], we assumed lower HCC levels in children with TD and a high level of comorbid conduct problems compared to children with TD but no conduct problems. For children with TD and a high level of emotional problem scores, we assumed higher HCC levels compared to children with TD without emotional problems. Furthermore, we hypothesised that psychotropic medication might lower HCC levels through symptom reduction.

## Methods

### Study design and participants

Our data are part of a large longitudinal European cohort, the European Multicenter Tics in Children Studies (EMTICS), which aims to identify genetic and environmental risk factors of TDs [32]. Only data from baseline measurements (first visit) were included in the present analysis. Children and their families were recruited from 16 child and adolescent psychiatry, neurology, and pediatric neurology outpatient clinics across Europe (for more details see [32]). The study was also advertised to patient organizations and other health professionals.

EMTICS comprised two separate cohort samples: COURSE and ONSET. For the COURSE sample, 715 children aged 3–16 years with an established diagnosis of Tourette syndrome or another chronic motor or vocal tic disorder according to DSM IV-TR criteria [1] were recruited. For the ONSET sample, 260 3–10-year-old first-degree relatives (siblings) of individuals with Tourette syndrome or another chronic tic disorder were recruited, who themselves never had tics, obsessive–compulsive disorder or trichotillomania.

Children with serious medical/neurological conditions, children who were treated with antibiotics in the past month, children of the ONSET sample with OCD or trichotillomania, and children of the COURSE study with a transient tic disorder were excluded. This resulted in a sample of 881 participants who met all inclusion criteria for this study (COURSE sample *N* = 663, ONSET sample *N* = 218). Use of medications (last 2 weeks) was documented by questionnaire (see Table 1). The study was approved by the Institutional Review Board of each participating center. Parents and their children provided written informed consent and assent as appropriate according to ethical regulations. While travel costs of participating families were reimbursed, no additional compensation was paid.

**Table 1:** Sample characteristics

	COURSE	ONSET	Test statistic	*p* value
	(*N* = 412)	(*N* = 131)		
Sex				
Male, *n* (%)	318 (77.2)	56 (42.7)	*X*^*2*^(1) = 54.99	*p* < .01
Female, *n* (%)	94 (22.8)	75 (57.3)		
Psychotropic medication				
No, *n* (%)	277 (67.2)	130 (99.2)	*X*^*2*^(1) = 54.23	*p* < .01
Yes, *n* (%)	135 (32.8)	1 (0.8)		
SDQ				
Emotional problem score, *M* (*SD*)	3.92 (2.59)	1.60 (1.86)	*t*(304.53) = 11.24	*p* < .01
Conduct problem score, *M* (*SD*)	2.53 (1.92)	1.39 (1.49)	*t*(278.59) = 7.09	*p* < .01
Age years, *M* (*SD*)	10.83 (2.77)	6.88 (2.13)	*t*(281.60) = 17.12	*p* < .01
PSS-P-10 parental report, *M* (*SD*)	17.58 (7.30)	10.45 (6.36)	*t*(541) = 10.03	*p* < .01
PSS-C-10 child report, *M* (*SD*)	17.61 (7.11)	–	–	–
YGTSS severity score, *M* (*SD*)	19.67 (8.47)	–	–	–
Medication				
First generation antipsychotics, *n* (%)	16 (3.9)	–	–	–
Second generation antipsychotics, *n* (%)	77 (18.7)	–	–	–
Serotonin reuptake inhibitors, *n* (%)	13 (3.2)	1 (0.8)	–	–
Other antidepressants, *n* (%)	1 (0.2)	–	–	–
ADHD medication, *n* (%)	42 (10.2)	–	–	–
Alpha agonists, *n* (%)	8 (1.9)	–	–	–
Mood stabilizers, *n* (%)	1 (0.2)	–	–	–
Benzodiazepines, *n* (%)	4 (1.0)	–	–	–

*SDQ* Strengths and Difficulties Questionnaire, *PSS-P-10* Perceived Stress Scale parental report (10-item scale), *PSS-C-10* Perceived Stress Scale child self-report (10-item scale), *YGTSS* Yale Global tic Severity Scale, *ADHD* attention/deficit-hyperactivity disorder

For PSS-C-10 N = 182

### Measures

#### HCC

As a physiological marker of longer-term stress, HCC was extracted from the three most proximal centimeters of the gathered scalp hair strains. As the growth rate of hair is approximately 1 cm per month, a cortisol exposure over the past 3 months is assumed to be indexed in these hair samples [14]. The analyzing procedure followed a protocol described in detail by Dettenborn and colleagues [33]. In a first step, each hair segment was washed for 3 min with 2.5 ml isopropanol in a 15-ml falcon tube on an overhead rotator and afterwards dried for at least 12 h. Commercial immunoassay with chemoluminesce detection (CLIA) from IBL-International/Tecan, Hamburg, Germany) were used for the quantification of cortisol levels in the hair samples. The analyzing procedure in our study differed from the procedure described by Dettenborn and colleagues [33] in two regards: (a) the hair was not pulverized for extraction and (b) only 7.5 mg (instead of 10 mg) hair was prepared. We took this approach on the base of results from extensive in-house experiments showing (a) that cortisol results do not differ when hair samples are ground before extraction or used in whole and (b) that a reliable detection of cortisol levels in hair extracts are already achieved from hair samples/segments of 4–7.5 mg mass. No significant increase in reliability nor absolute concentration is achieved when cortisol is extracted from hair segments with a larger hair mass. At the same time, taking a smaller amount of hair enhances the acceptance of the hair sampling procedure, especially in samples of children.

At baseline, we collected 694 hair samples from approx. 2 cm below the cranial bone (COURSE sample *N* = 520; ONSET sample *N* = 174). Due to insufficient weight (≤ 4 mg) or insufficient length (≤ 2 cm) 112 hair samples were excluded. Another three hair samples were excluded due to implausible hair cortisol values (≥ 43.88 pg/mg; + 3 SD). This resulted in a total hair sample size of 579 (COURSE sample *N* = 436; ONSET sample *N* = 143).

#### Perceived stress scale (PSS-10)

For a subjective measure of perceived stress, the well-established *Perceived Stress Scale* [34] was used. Participants indicated on a five-point response scale how often stressful life situations had occurred over the past month. The PSS-P-10 parental report was collected for all participating children. For children aged 11 and above, child self-report (PSS-C-10) was additionally collected (182 individuals of the COURSE sample). For 32 participants with valid hair samples, PSS-P-10 data were incomplete.

#### Yale global tic severity scale (YGTSS)

To assess tic severity, we used the clinician-rated *Yale Global tic Severity Scale* (YGTSS [35]). This scale evaluates the frequency and intensity as well as interpersonal and academic impairment caused by the tics over the previous week. The sum score for the severity of motor and vocal tics (Total Tic Severity Score, maximum score = 50) was used for the present analyses. YGTSS was collected for all participants with TDs. For all participants of the COURSE sample with valid hair samples, YGTSS data were complete.

#### Strengths and difficulties questionnaires (SDQ)

The *Strengths and Difficulties Questionnaires* (SDQ; Goodman, 36) was used to assess emotional and behavioral problems, rated by the parents. The SDQ consists of 25 items comprising five domains (emotional problems, conduct problems, hyperactivity and inattention, peer relationship problems and prosocial behavior). For the present analyses, the subscale scores of ‘emotional problems’ (five items, for example: ‘Many worries or often seems worried’, ‘Often unhappy, depressed or tearful’, ‘Many fears, easily scared’, range 0–10) and ‘conduct problems’ (five items, for example: ‘Often loses temper’, ‘Generally well behaved, usually does what adults request’, ‘Often fights with other children or bullies them’, range 0–10) were used. To examine the influence of emotional and behavior problems on HCC, the raw sum subscale scores of ‘emotional problems’ and ‘conduct problems’ were dichotomized using cut-off values above the 90% percentiles (emotional problems > 4; conduct problems > 3) [36, 37]. For 15 participants with valid hair samples, SDQ data were incomplete.

### Statistical analysis

To identify differences in sex and prescribed psychotropic medication between the COURSE and ONSET samples, we conducted chi-square tests. Group differences in age, the emotional and conduct problem scores of the SDQ and perceived stress were examined by independent t-tests.

Due to skewed distribution of HCC data, we applied a Box–Cox transformation [38] with − 0.1 as best fitting lambda. Transformed HCC values were used for all analysis. We investigated group differences in HCC between the COURSE and ONSET samples by Analysis of Variance (ANOVA) with HCC as dependent variable and group (COURSE sample, ONSET sample) and sex (male, female) as independent variables and age as a covariate. There was a large age difference between our two samples. To reduce potential confounding effects on differences in HCC, we performed a 2:1 case–control matching for age with a match tolerance of 0.65, resulting in reduced samples (COURSE sample: *N* = 119, ONSET sample: *N* = 61).

To identify relationships between HCC, tic severity, and perceived psychosocial stress (parental report and child self-report), Pearson correlations were conducted within the COURSE sample. Alpha levels were Bonferroni-adjusted (0.05/4 = 0.0125). In addition, partial correlations were calculated with potential confounders (age, sex, medication, emotional and conduct problem score). The associations between perceived stress and tic severity are only of secondary interest to this paper (and will be addressed in more detail in a prospective EMTICS report).

To examine potential influences of sex, psychotropic medication and emotional and behavioral problems on HCC levels in patients with TDs, ANCOVAs were conducted (within the COURSE sample). HCC was included as dependent variable, sex (male, female), psychotropic medication status (no, yes), SDQ categorization (> P90, < P90) for the emotional problem scale and conduct problem scale as independent variables, and age as a covariate. The model further included interaction terms of the independent variables.

## Results

At baseline, valid hair samples were available for 579 children (COURSE sample: *N* = 436, ONSET sample: *N* = 143). Due to missing data of the PSS-P-10 and SDQ, data of 543 participants were used for analysis (COURSE sample: *N* = 412; ONSET sample: *N* = 131; see Table 1 for sample characteristics).

73 children in the COURSE sample and 15 children in the ONSET sample were taking medication that could potentially confound cortisol levels, e.g. corticosteroid-containing medication. We tested whether those medications had an effect on HCC in our study, but found no difference between HCC of children, who did take medication that could potentially confound cortisol and those who did not (neither in the COURSE, nor in the ONSET sample). Moreover, our study results remained the same, when we excluded those children for test purposes from the analyses.

### Differences in HCC between children and adolescents with TDs and unaffected siblings

With the age-matched sample, ANOVAs were performed (COURSE sample: *N* = 119, 25 female; ONSET sample: *N* = 61, 36 female). There was no significant difference in HCC between the COURSE and ONSET samples (*F*(1,176) = 2.67, *p* = 0.10, partial *η*^*2*^ = 0.015) or between males and females (*F*(1,176) = 0.92, *p* = 0.34, partial *η*^*2*^ = 0.005). Furthermore, no interaction effect of group and sex on HCC (*F*(1,176) = 0.25, *p* = 0.62, partial *η*^*2*^ = 0.001) was found.

### Relationship between HCC, tic-severity and perceived psychosocial stress

Within the COURSE sample, results of bivariate correlations showed no association between HCC and parental report of the PSS-10 (*r*(412) = −0.05, *p* = 0.34) as well as between HCC and children’s self-report of the PSS-10 (*r*(182) = 0.13, *p* = 0.08). We did not find any association between HCC and tic severity (*r*(412) = −0.01, *p* = 0.92). The parental report of the PSS-10 was positively associated with tic severity (*r*(412) = 0.29, *p* < 0.0125). A similar relationship was found between children’s self-report of the PSS-10 and tic severity (*r*(182) = 0.20, *p* < 0.0125).

Partial correlation with possible confounders (age, sex, medication, emotional and conduct problems) did not change the results (see Table 3 in the supplemental material).

### Interaction of HCC with emotional and behavioral problems and psychotropic medication use within individuals with TDs

Within the COURSE sample, there was no significant effect of sex (*F*(1,395) = 3.44, *p* = 0.07, partial *η*^*2*^ = 0.009), psychotropic medication (*F*(1,395) = 1.85, *p* = 0.18, partial *η*^*2*^ = 0.005), emotional problems (*F*(1,395) = 0.41, *p* = 0.52, partial *η*^*2*^ = 0.001) or conduct problems (*F*(1,395) = 0.52, *p* = 0.47, partial *η*^*2*^ = 0.001) on HCC, co-varying for age (*F*(1,395) = 0.56, *p* = 0.46, partial *η*^*2*^ = 0.001; for means and standard deviations see Table 2). No interaction effects were found.

**Table 2:** Means of untransformed HCC separated for sample characteristics within the COURSE sample

	HCC (pg/mg) *M* (*SD*)
Sex	
Male, *n* = 318	3.26 (4.29)
Female, *n* = 94	3.47 (4.85)
Psychotropic medication	
No, *n* = 277	3.34 (4.65)
Yes, *n* = 135	3.23 (3.93)
SDQ emotional problem scale	
> P90, *n* = 162	2.96 (3.49)
< P90, *n* = 250	3.52 (4.92)
SDQ conduct problem scale	
> P90, *n* = 116	2.95 (3.14)
< P90, *n* = 296	3.44 (4.83)

*HCC* Hair Cortisol Concentration, *SDQ* Strengths and Difficulties Questionnaire

## Discussion

The primary focus of this study was to investigate the role of HCC as a physiological measure of longer-term stress levels in children and adolescents with TDs and unaffected siblings of individuals with TDs. We found no elevated HCC levels in individuals with TDs. Furthermore, tic severity or perceived psychosocial stress was not related to HCC levels in children and adolescents with TDs. This is against our expectation but is in line with some previous findings. For example, a meta-analysis of 66 studies with 124 divers subsamples [15] showed no consistent association between HCC and perceived stress. However, a positive correlation was found between tic severity and both self- and parent report of perceived psychosocial stress. Neither sex, psychotropic medication intake, nor emotional and conduct problems had a significant effect on HCC levels in children and adolescents with TDs.

Results of the present study demonstrated that HCC as a physiological marker of chronic stress did not correspond to results from previous studies using physiological markers of acute stress, which found stronger activation of the HPA axis in patients with TDs as assessed by salivary cortisol, ACTH in the blood plasma, and CRH in the cerebrospinal fluid [11–13]. Previous studies used prospective measures of stress, i.e. captured the diurnal activation of the HPA axis, while we assessed HCC, which is a retrospective measure of longer-term cortisol secretion and accumulation. Moreover, previous studies assessed activation of the HPA axis in patients with TDs in response to external, single stressors (such as a mock MRI scan or lumbar puncture), while we aimed to describe the relationship between longer-term stress in daily life and tics themselves.

Previous studies on HCC levels, demonstrated different patterns in mental disorders. While for some mental disorders, increased HCC levels were found (e.g. major depressive disorder; [17]), for other mental disorders, decreased HCC levels were reported (e.g. anxiety [20, 39]). In some mental disorders, HCC levels seem to fluctuate over time (e.g. post-traumatic disorder [40]), while in other mental disorders, age of onset seems to influence changes in HCC levels (e.g. bipolar disorder [41]). It has been argued that changes in activation of the HPA axis related to mental disorders are probably more subtle with smaller effect sizes than changes of HCC in relation to chronic life stressors, as experienced by endurance athletes, shift workers and unemployed individuals [42]. In a meta-analysis, it has been argued that the relationship between chronic stress and HPA activity might depend on the intensity of the stressful event and hence fluctuate over time, with higher levels of HPA activity during the period of the stressful event and a long-term decline even below normal levels [43]. This mediation by stress intensity and fluctuation over time might explain why in the present cross-sectional data HCC was not associated with severity of tics nor with perceived stress. This might also explain why HCC was not increased in participants with TDs compared to siblings of individuals with TDs, who themselves never had tics. In a recent study with 37 couples with no history of psychiatric disorders, self-reported occurrence of weekly hassles (as assessed with the Weekly Hassle Scale) was associated with HCC with a time lag of about 4 weeks, while self- and partner reports of the perceived stress scale (PSS-10) or the Trier Inventory of Chronic Stress showed no association with HCC [44]. The authors concluded that psychosocial stress levels had the highest correlation with HCC, if they were based on self-reports of the weekly hassles and assessed between 3 and 8 weeks before hair sampling. It is, therefore, possible that the aspect of psychosocial stress measures on the PSS does not correlate as closely with HCC as that assesses on the Weekly Hassle Scale. Furthermore, collecting data of HCC in the three most proximal centimeters of the scalp hair (refers to the cortisol exposure over the past 3 months), tic severity (refers to the past week), and perceived psychosocial stress (refers to the past 4 weeks) at the same time point could also explain why we did not find a relationship of HCC with tic severity and perceived stress, as psychosocial stress levels at the time of assessment may already have changed. The longitudinal data of EMTICS will provide more insight into the time lag in the relationship of HCC, tic severity and perceived stress.

Finally, as a large proportion of children and adolescents with TDs is also affected by emotional and behavioral problems and take psychotropic medication for symptom reduction, we were interested in how emotional and behavioral problems as well as psychotropic medication might affect levels of HCC as a physiological marker of longer-term stress. Against our expectations, we found no significant effect of emotional or behavioral problems on HCC levels in the present data. However, previous findings demonstrated that even in non-clinical populations behavioral problems are associated with basal cortisol (not as a reactivity to a stressor) and this association is moderated by age [29]. Different from what we expected, we also did not find a significant effect of psychotropic medication on HCC levels in the present data. Several possible explanations might account for the missing link. For example, patients who are on psychotropic medication might have suffered from stronger symptoms and higher stress levels in the first place, making medication intake necessary. Medication intake might have then reduced symptoms and stress to a level that is comparable to those patients, who are not in need for medication. One might also speculate that the missing effect might trace back to non-responders, whose inadequate response to the medication actually increased rather than decreased stress. Moreover, given the very wide range of psychotropic medications, it is also plausible that a consistent effect on HCC is not existent.

Several limitations should be acknowledged. First, there was a significant age and sex difference between the participants with TDs and unaffected sibling of individuals with TDs. Consequently, analysis of a matched sample led to a significant reduction in sample size. Second, since PSS-10 as a self-report could only be obtained in adolescents older than 11 years of age, this measurement was only available for a part of the participants with TDs. Third, the HCC levels of children with TDs were compared with an at-risk group (siblings of children with TDs). A healthy control group without risk was not assessed.

Summarizing, the present study could not reveal a relationship between HCC and TDs. Future studies should investigate how waxing and waning of tics relate to fluctuations in perceived stress and HCC. Furthermore, diurnal cortisol levels (e.g. measured by saliva cortisol) and longer-term cortisol levels (measured by HCC) should be investigated congruently and longitudinally in individuals with TDs and related to short-term acute stressors as well as longer-term stress in daily life (e.g. daily hassles). Emotional and behavioral problems should be considered.

## Supplementary Information

Below is the link to the electronic supplementary material.

Supplementary file1 (DOCX 15 KB)

## Data Availability

Not applicable.

## Footnotes

1 The term Tic disorder (TD) is used to cover all tic disorders including Tourette Syndrome—if nothing else is explicitly stated.

## References

[1] American Psychiatric-Association (2000) Diagnostic and statistical manual of mental disorders DSM-IV-TR (Text revision) (4). Washington

[2] Knight T, Steeves T, Day L, Lowerison M, Jette N, Pringsheim T (2012). Prevalence of tic disorders: a systematic review and meta-analysis. PediatrNeurol.

[3] Robertson MM (2008). The prevalence and epidemiology of Gilles de la Tourette syndrome. Part 2: tentative explanations for differing prevalence figures in GTS, including the possible effects of psychopathology, aetiology, cultural differences, and differing phenotypes. J Psychosom Res.

[4] Dietrich A, Fernandez TV, King RA, State MW, Tischfield JA, Hoekstra PJ, Heiman GA, TIC Genetics Collaborative Group (2015). The Tourette International Collaborative Genetics (TIC Genetics) study, finding the genes causing Tourette syndrome: objectives and methods. Eur Child Adolesc Psychiatry.

[5] Grados MA (2010). The genetics of obsessive-compulsive disorder and Tourette syndrome: an epidemiological and pathway-based approach for gene discovery. J Am Acad Child Adolesc Psychiatry.

[6] Pagliaroli L, Vető B, Arányi T, Barta C (2016). From genetics to epigenetics: new perspectives in Tourette syndrome research. Front Neurosci.

[7] Buse J, Kirschbaum C, Leckman JF, Münchau A, Roessner V (2014). The modulating role of stress in the onset and course of Tourette’s syndrome: a review. BehavModif.

[8] Hoekstra PJ, Anderson GM, Limburg PC, Korf J, Kallenberg CGM, Minderaa RB (2004). Neurobiology and neuroimmunology of Tourette’s syndrome: an update. Cell Mol Life Sci.

[9] Hoekstra PJ, Dietrich A, Edwards MJ, Elamin I, Martino D (2012). Environmental factors in Tourette syndrome. NeurosciBiobehav Rev.

[10] Robertson MM, Eapen V (2017). The psychosocial aspects of the Gilles de la Tourette syndrome: empirical evidence from the literature. CurrBehavNeurosci Rep.

[11] Corbett BA, Mendoza SP, Baym CL, Bunge SA, Levine S (2008). Examining cortisol rhythmicity and responsivity to stress in children with Tourette syndrome. Psychoneuroendocrinology.

[12] Chappell P, Riddle M, Anderson G, Scahill L, Hardin M, Walker D, Leckman J (1994). Enhanced stress responsivity of Tourette syndrome patients undergoing lumbar puncture. BiolPsychiat.

[13] Chappell P, Leckman J, Goodman W, Bissette G, Pauls D, Anderson G, Cohen D (1996). Elevated cerebrospinal fluid corticotropin-releasing factor in Tourette’s syndrome: comparison to obsessive compulsive disorder and normal controls. BiolPsychiat.

[14] Stalder T, Kirschbaum C (2012). Analysis of cortisol in hair—state of the art and future directions. Brain BehavImmun.

[15] Stalder T, Steudte-Schmiedgen S, Alexander N, Klucken T, Vater A, Wichmann S, Miller R (2017). Stress-related and basic determinants of hair cortisol in humans: a meta-analysis. Psychoneuroendocrinology.

[16] Dettenborn L, Tietze A, Bruckner F, Kirschbaum C (2010). Higher cortisol content in hair among long-term unemployed individuals compared to controls. Psychoneuroendocrinology.

[17] Dettenborn L, Muhtz C, Skoluda N, Stalder T, Steudte S, Hinkelmann K, Otte C (2012). Introducing a novel method to assess cumulative steroid concentrations: increased hair cortisol concentrations over 6 months in medicated patients with depression. Stress (Amsterdam, Netherlands).

[18] Kirschbaum C, Tietze A, Skoluda N, Dettenborn L (2009). Hair as a retrospective calendar of cortisol production-increased cortisol incorporation into hair in the third trimester of pregnancy. Psychoneuroendocrinology.

[19] Steudte S, Kirschbaum C, Gao W, Alexander N, Schönfeld S, Hoyer J, Stalder T (2013). Hair cortisol as a biomarker of traumatization in healthy individuals and posttraumatic stress disorder patients. BiolPsychiat.

[20] Steudte S, Stalder T, Dettenborn L, Klumbies E, Foley P, Beesdo-Baum K, Kirschbaum C (2011). Decreased hair cortisol concentrations in generalised anxiety disorder. Psychiatry Res.

[21] Van Uum SHM, Sauvé B, Fraser LA, Morley-Forster P, Paul TL, Koren G (2008). Elevated content of cortisol in hair of patients with severe chronic pain: a novel biomarker for stress. Stress (Amsterdam, Netherlands).

[22] Golub Y, Kuitunen-Paul S, Panaseth K, Stonawski V, Frey S, Steigleder R, Eichler A (2019). Salivary and hair cortisol as biomarkers of emotional and behavioral symptoms in 6–9 year old children. PhysiolBehav.

[23] Schloß S, Ruhl I, Müller V, Becker K, Skoluda N, Nater UM, Pauli-Pott U (2018). Low hair cortisol concentration and emerging attention-deficit/hyperactivity symptoms in preschool age. Dev Psychobiol.

[24] Kurlan R, Como PG, Miller B, Palumbo D, Deeley C, Andresen EM, McDermott MP (2002). The behavioral spectrum of tic disorders: a community-based study. Neurology.

[25] Bornstein RA, Stefl ME, Hammond L (1990). A survey of Tourette syndrome patients and their families: the 1987 Ohio Tourette survey. J Neuropsychiatry ClinNeurosci.

[26] Buse J, Enghardt S, Kirschbaum C, Ehrlich S, Roessner V (2016). Tic frequency decreases during short-term psychosocial stress—an experimental study on children with tic disorders. Front Psychiatry.

[27] Kraft JT, Dalsgaard S, Obel C, Thomsen PH, Henriksen TB, Scahill L (2012). Prevalence and clinical correlates of tic disorders in a community sample of school-age children. Eur Child Adolesc Psychiatry.

[28] Freeman RD, Fast DK, Burd L, Kerbeshian J, Robertson MM, Sandor P (2000). An international perspective on Tourette syndrome: selected findings from 3,500 individuals in 22 countries. Dev Med Child Neurol.

[29] Alink LR, Van Ijzendoorn MH, Bakermans-Kranenburg MJ, Mesman J, Juffer F, Koot HM (2008). Cortisol and externalizing behavior in children and adolescents: mixed meta-analytic evidence for the inverse relation of basal cortisol and cortisol reactivity with externalizing behavior. Dev Psychobiol.

[30] Lopez-Duran NL, Kovacs M, George CJ (2009). Hypothalamic— pituitary—adrenal axis dysregulation in depressed children and adolescents: a meta-analysis. Psychoneuroendocrinology.

[31] Ruttle PL, Shirtcliff EA, Serbin LA, Fisher DB, Stack DM, Schwartzman AE (2011). Disentangling psychobiological mechanisms underlying internalizing and externalizing behaviors in youth: longitudinal and concurrent associations with cortisol. HormBehav.

[32] Schrag A, Martino D, Apter A, Ball J, Bartolini E, Benaroya-Milshtein N, EMTICS Collaborative Group (2019). European Multicentre Tics in Children Studies (EMTICS): protocol for two cohort studies to assess risk factors for tic onset and exacerbation in children and adolescents. Eur Child Adolesc Psychiatry.

[33] Dettenborn L, Tietze A, Kirschbaum C, Stalder T (2012). The assessment of cortisol in human hair: associations with sociodemographic variables and potential confounders. Stress.

[34] Cohen S, Kamarck T, Mermelstein R (1983). A global measure of perceived stress. J Health SocBehav.

[35] Leckman JF, Riddle MA, Hardin MT, Ort SI, Swartz KL, Stevenson J, Cohen DJ (1989). The Yale Global Tic Severity Scale: initial testing of a clinician-rated scale of tic severity. J Am Acad Child Adolesc Psychiatry.

[36] Goodman R (1997). The strengths and difficulties questionnaire: a research note. J Child Psychol Psychiatry.

[37] Goodman R (2001). Psychometric properties of the strengths and difficulties questionnaire. J Am Acad Child Adolesc Psychiatry.

[38] Osborne JW (2010). Improving your data transformations: applying the box-cox transformation. Pract Assess Res Eval.

[39] Straub J, Klaubert LM, Schmiedgen S, Kirschbaum C, Goldbeck L (2017). Hair cortisol in relation to acute and post-traumatic stress symptoms in children and adolescents. Anxiety Stress Coping.

[40] Luo H, Hu X, Liu X, Ma X, Guo W, Qiu C, Li T (2012). Hair cortisol level as a biomarker for altered hypothalamic-pituitary-adrenal activity in female adolescents with posttraumatic stress disorder after the 2008 Wenchuan earthquake. BiolPsychiat.

[41] Manenschijn L, Spijker AT, Koper JW, Jetten AM, Giltay EJ, Haffmans J, van Rossum EFC (2012). Long-term cortisol in bipolar disorder: associations with age of onset and psychiatric co-morbidity. Psychoneuroendocrinology.

[42] Staufenbiel SM, Penninx BWJH, Spijker AT, Elzinga BM, van Rossum EFC (2013). Hair cortisol, stress exposure, and mental health in humans: a systematic review. Psychoneuroendocrinology.

[43] Miller GE, Chen E, Zhou ES (2007). If it goes up, must it come down? Chronic stress and the hypothalamic-pituitary-adrenocortical axis in humans. Psychol Bull.

[44] Weckesser LJ, Dietz F, Schmidt K, Grass J, Kirschbaum C, Miller R (2019). The psychometric properties and temporal dynamics of subjective stress, retrospectively assessed by different informants and questionnaires, and hair cortisol concentrations. Sci Rep.

