# Supplementary material for: Hair cortisol-a stress marker in children and adolescents with chronic tic disorders? A large European cross-sectional study
Source: Eur Child Adolesc Psychiatry. 2021 Jan 18;31(5):771–9. doi: 10.1007/s00787-020-01714-1 (PMC9142457; doi:10.1007/s00787-020-01714-1)
Supplement: Supplementary file 1 — Supplementary file1 (DOCX 15 KB) [file 787_2020_1714_MOESM1_ESM.docx]

**Supplementary Material**

Table 3. Correlation coefficient (and p value) of partial correlation with possible confounders

| Confounder |  | PSS-P-10  (parental-report) | PSS-C-10  (child's self-report) | YGTSS  Severity Score |
| --- | --- | --- | --- | --- |
| Age | HCC | .04 (.39) | .12 (.12) | -.01 (.84) |
|  | PSS-P-10 | - | .55 (.00) | .28 (.00) |
|  | PSS-C-10 | - | - | .20 (.01) |
| Sex | HCC | .04 (.38) | .11 (.14) | .00 (.99) |
|  | PSS-P-10 | - | .53 (.00) | .34 (.00) |
|  | PSS-C-10 | - | - | .21 (.01) |
| Psychotropic medication use | HCC | .04(.39) | .13 (.09) | -.01 (.80) |
|  | PSS-P-10 | - | .54 (.00) | .27 (.00) |
|  | PSS-C-10 | - | - | .18 (.02) |
| SDQ Conduct Problem Score | HCC | .07 (.14) | .20 (.01) | .00 (.95) |
|  | PSS-P-10 | - | .48 (.00) | .31 (.00) |
|  | PSS-C-10 | - | - | .17 (.02) |
| SDQ Emotional Problem Score | HCC | .07 (.19) | .17 (.03) | .00 (.99) |
|  | PSS-P-10 | - | .41 (.00) | .23 (.00) |
|  | PSS-C-10 |  |  | .08 (.30) |

*Note.* SDQ = Strengths & Difficulties Questionnaire; PSS-10 = Perceived Stress Scale (10-item scale); YGTSS = Yale Global tic Severity Scale. N = 412 for HCC, PSS-10 (parental-report) and YGTSS; N = 182 for PSS-10 (child's self-report).
